# High origin of dorsal branch of the ulnar nerve and variations in its branching pattern and distribution: a case report

**DOI:** 10.1186/1757-1626-2-9130

**Published:** 2009-12-02

**Authors:** Polly Lama, Bhagath K Potu, Kumar MR Bhat

**Affiliations:** 1 Department of Anatomy, Sikkim Manipal University, Gangtok, Sikkim, India; 2 Department of Anatomy, Kasturba Medical College, Manipal University, Manipal, India

## Abstract

**Introduction:**

Ulnar nerve is a branch of the brachial plexus. In the front of the forearm, normally near the wrist joint, it gives a dorsal cutaneous branch which supplies the skin of the dorsum of the hand.

**Case presentation:**

The present case reports a very rare finding, the dorsal branch of the ulnar nerve along with the main nerve trunk originated between the two heads of the flexor carpi ulnaris muscle, after descending along the medial border of the forearm extensor surface, on the dorsal aspect of the wrist it is divided into three branches, one medial and two lateral. The medial most division received a communicating branch from the superficial ramus of the ulnar nerve and continued as the medial proper digital nerve of the little finger. The lateral two divisions became cutaneous on the medial half of the dorsum of the hand along the medial three digits i.e. radial and ulnar side of little, ring and middle finger.

**Conclusion:**

The site, extent of injury, variations and the delay in the treatment, significantly influences the outcome of ulnar nerve repair. Thus, an adequate knowledge of all possible variations in the ulnar nerve may be important for clinicians and may help to explain uncommon symptoms.

## Introduction

Normally, the ulnar nerve arises from the medial cord of the brachial plexus runs distally through the axilla medial to the axillary artery and continues distally medial to the brachial artery as far as midarm, here it pierces the middle intermuscular septum and appears between the medial epicondyle and olecranon process to enter the forearm between two heads of the flexor carpi ulnaris muscle and descends along the medial side of the front of the forearm. About 5 cm proximal to the wrist it gives of a dorsal branch which is responsible for the sensory innervations to the medial one and a half digit [1].

The variation in the branching pattern of the ulnar nerve in the forearm is very rare and may be important for the diagnosis of unexpected and uncommon clinical conditions. One such very rare variation is "all ulnar hand" where both motor and sensory nerve supply of the hand is by the ulnar nerve without any communication with either median or radial nerve [2]. Rarely, ulnar nerve may have Marinacci or reverse Martin-Gruber communication with the median nerve in the distal forearm [3].

The branching pattern of the dorsal ulnar nerve is also important while exploring the orthoscopic portals [4]. The higher origin of the dorsal branch makes it liable to superficial injuries and laceration in the forearm region. Documentation of such variation will also contribute to the knowledge of anatomy of the ulnar nerve and such releases is necessary for surgeons operating on the dorsal aspect making them aware of such anomalies would help check any inadvertent injuries during surgical procedures.

## Case Presentation

**Figure 1:**
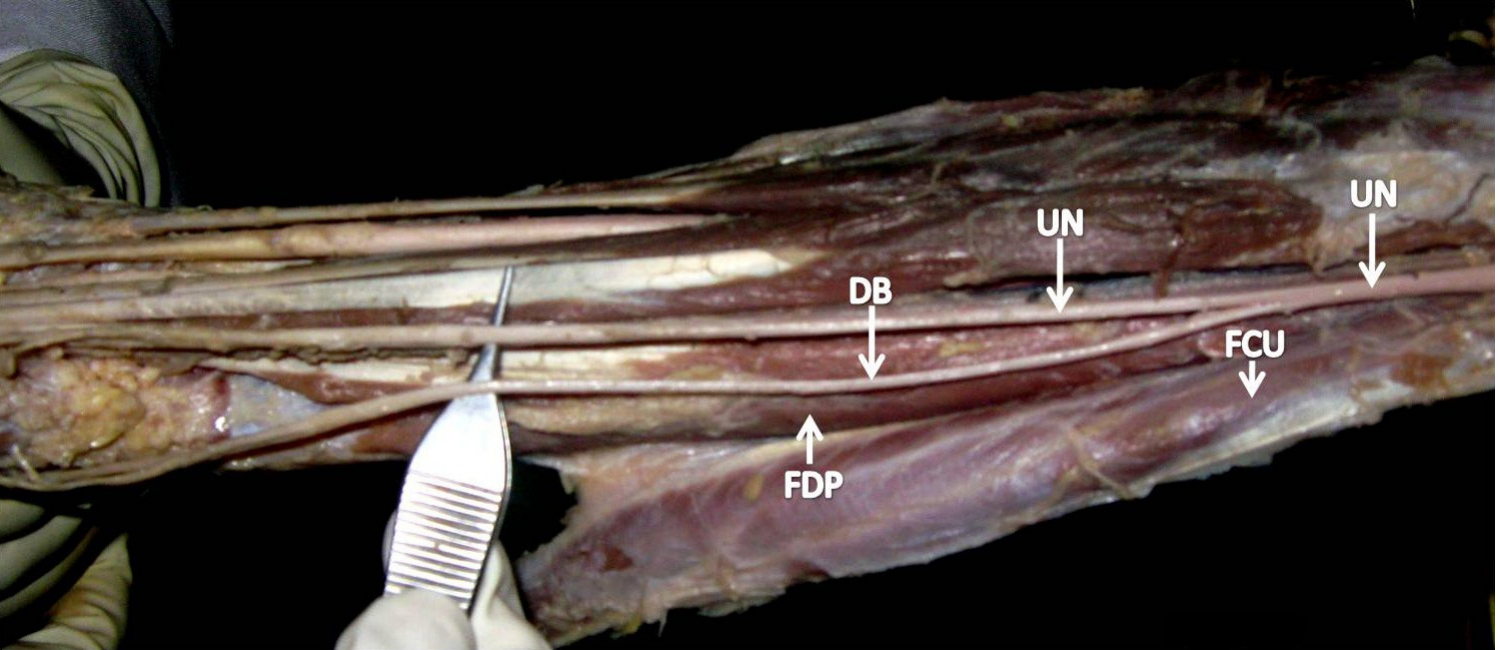
**Ulnar nerve gives a dorsal branch high in the forearm between Flexor carpi ulnaris muscle and Flexor digitorum profundus muscle near the cubital region**.

**Figure 2:**
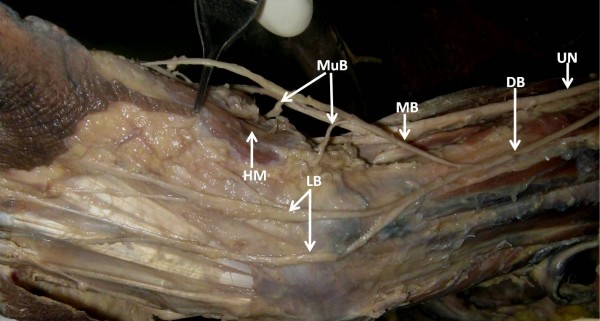
**The dorsal branch of the unlar nerve then divides into one medial branch which enters the palm and lateral branches which enters the dorsum of the hand**. The medial branch further gives muscular branches to hypothenar muscles.

**Figure 3:**
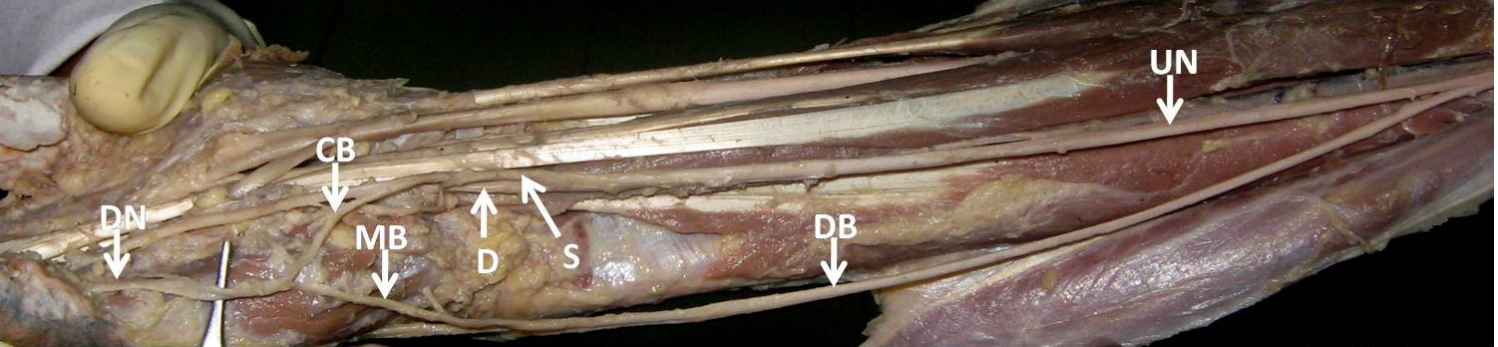
**Near the wrist the ulnar nerve divides into superficial and deep branches**. Superficial division further gives a communicating branch which joins the medial branch of the dorsal ulnar nerve and continues as medial proper digital nerve of the little finger.

**Figure 4:**
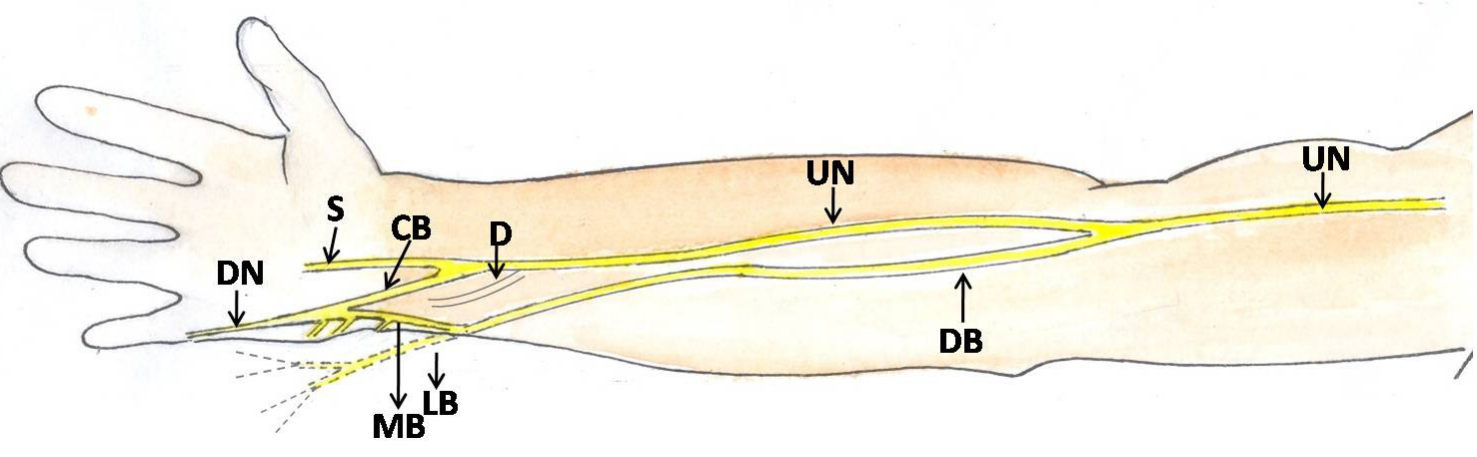
**Schematic diagram to show the branching pattern of the doral branch of the ulnar nerve in the forearm, wrist and in the hand**.

During a routine cadaveric dissection of the forearm a rare variation was encountered on the right side of a 38 year male cadaver in Department of Anatomy, Sikkim-Manipal University, Gangtok, Sikkim. The forearm and palm was exposed and the fascia was carefully removed. After the reflection of the flexor carpi ulnaris medially and removal of the flexor retniaculum, the main trunk of the Ulnar nerve was identified. After its course in the forearm, the ulnar nerve entered the Guyon's canal in the hand underneath the flexor retniaculum and divided into superficial (sensory) and deep (motor) branches. The dorsal branch of ulnar nerve (dorsal ulnar nerve) originated from the ulnar nerve trunk at the level of the elbow near the cubital fossa approximately at the junction of upper one fourth and the lower three fourth (Figure 1). It descended down initially under and then along the medial border of the flexor carpi ulnaris in the forearm. After emerging out from the flexor carpi ulnaris muscle and before turning on to the dorsal part of the forearm, the dorsal branch, divided into three branches, one medial and two lateral (Figure 2). The medial most branch emerged about 4 cm proximal to the styloid process of the ulna and gave off few thin branches along its course on the hypothenar eminence to the abductor digiti minimi muscle and to the skin around it. Then this medial branch merged with the communicating branch from the superficial palmar branch of the ulnar nerve and continued as the medial proper digital nerve to the little finger (Figure 3 &4). The lateral two divisions of the dorsal branch of the ulnar nerve became cutaneous and were supplying the medial half of the dorsum of hand along the medial three digits i.e. radial and ulnar side of little, ring and middle finger. The branching pattern and course of the ulnar nerve on the left side of the body was normal.

## Discussion

Anomalies of the sensory innervations of the hand are uncommon [5]. The knowledge of the nerve variation is important and could explain the unexpected sensory/motor loss or pain in the patients following surgical procedure or trauma. As the name suggests, the dorsal branch is designated to supply the dorsal aspect of the forearm and hand but many cases have been reported in the past to show its innervations even to the ventral aspect of the forearm and hand including hypothenar muscles.

The present case revealed a higher origin of the dorsal branch of ulnar nerve in the forearm and its termination by uniting with a communicating branch from the superficial ramus of ulnar nerve and its subsequent continuation as medial proper digital branch of little finger is rare. Bergman and his group [6] and Bozkurt and co-worker [7] have described the medial proper digital nerve to the little finger arising from the dorsal branch of the ulnar nerve and the dorsal branch of the ulnar nerve to have a higher origin. The entire little finger is shown to supplied by the digital branches from the dorsal branch of the ulnar nerve [8]. In 2005, Paul and his team [9] also found a higher origin of ulnar nerve in the forearm and its connection with the deep branch of the ulnar nerve. In 1963 itself Kaplan [10] descried a nerve branch that arose from the dorsal cutaneous branch of the ulnar nerve and finally merged with the superficial ramus of the ulnar nerve, this type of communication has been termed as "Kaplan's anastomosis". Hoogbergen and Kauer [11] found a significant case of Kaplan anastomosis, the dorsal cutaneous branch emerged approximately 2.5 cm proximal to the ulnar styloid process gave off three branches and eventually merged with the deep ramus on ulnar nerve. Georgis and his team [12] reported a case where the dorsal cutaneous nerve was united with the trunk of the ulnar nerve before its bifurcation into superior and deep ramus. Standring [1] has stated that the innervations of the hypothenar muscles of the palm are usually by the deep terminal branches of ulnar nerve in the palm. In the present study we detected a branch from the dorsal branch to the hypothenar muscles. However, similar innervations were reported rarely [9,13]. The variant digital branches from the ulnar nerve to the ulnar half of the index, ulnar half of the middle, and radial half of the ring finger without any communication between these digital branches and that of the median nerve has also been reported [14].

This finding is important as in case of any injury to the deep terminal ramus of the ulnar nerve as the extra supply from dorsal branch may spare the muscles in the hypothenar eminence. Knowing the existence of the abnormal communication between the dorsal branch and the superficial terminal branch is very essential, as it may be damaged during surgery, in trauma in these area and in the condition which involve Guyon's canal [12]. It has also been indicated that the congenital abnormal course of the ulnar nerve branches in the forearm may involved in compressive ulnar neuropathy and can be corrected with surgical intervention [15]. As the dorsal branch of the ulnar nerve passes close to the 6 Radial portal used in wrist arthroscopy, the knowledge about the possible variations in the course of this nerve is helpful in avoiding the iatrogenic injury to the nerve during arthroscopy [16]. Finally, the knowledge of the variations in the dorsal branch of ulnar nerve is important in electrodiagnosis [17] and may be significant while using forearm flap, nerve block, carpel tunnel release, reconstruction of ulnar bone and other surgical interventions of the forearm and the hand and may help the hand surgeons to interpret discrepancies in sensory loss after either dorsal or palmar injuries.

## List of Abbreviations

UN: Ulnar Nerve; DB: Dorsal Bracnh; FCU: Flexor Carpi Ulnaris; FDP: Flexor Digitorum Profundus; MB: Medial Branch; LB: Lateral Branch; HM: Hypothenar Muscles; S: Superficial; D: Deep; CB: Communicating Branch; DN: Digital Nerve.

## Consent

Written informed consent was obtained from the concerned authorities for publication of this case report. A copy of the written consent is available for review by the Editor-in-Chief of this journal.

## Competing interests

The authors declare that they have no competing interests.

## Authors' contributions

PL found this variation, photographed and did the literature survey, BKP helped in manuscript preparation and KMB prepared the figures and wrote the case report. All authors had gone through the final manuscript and approved it.
